# Supplementary material for: A Likelihood Approach for Real-Time Calibration of Stochastic Compartmental Epidemic Models
Source: PLoS Comput Biol. 2017 Jan 17;13(1):e1005257. doi: 10.1371/journal.pcbi.1005257 (PMC5240920; doi:10.1371/journal.pcbi.1005257)
Supplement: S1 File — (TAR.GZ) [file pcbi.1005257.s014.tar.gz › HSPH_Online-SI-Revision/IPoi09/n100-extreme/n100-extreme_BENCH_table-8.pdf]

1	R0	{2.76052, 16.3913}	{2.43563, 12.1747}	{{2.00488, 8.68514}, {2.86118, 15.5722}}	{12.8828, 25.7245}	inf	695.	552.662	{46., 1338.}	68.6513	0.51271	newly	417.	497.352	{30., 1119.}	89.6241	0.465514	Reff	2.73137	2.45027	{2.12039, 2.75552}	11.2926	ar	92090.	88083.4	{83156., 92951.}	4.62031
2	R0	{2.18202, 15.1734}	{1.7889, 8.5897}	{{1.68297, 7.5059}, {1.93816, 10.0503}}	{18.0165, 43.3898}	inf	2011.	6895.73	{1772., 11534.}	252.386	0.507675	newly	1160.	6396.25	{1837., 9062.}	455.557	0.692132	Reff	2.10076	1.41902	{1.0872, 1.75258}	32.4521	ar	84264.	72178.3	{67865., 77879.}	14.3427
3	R0	{2.57891, 12.5971}	{2.77516, 13.9889}	{{2.45839, 11.6881}, {2.96947, 15.6207}}	{10.2577, 15.1351}	inf	340.	792.358	{104., 1558.}	160.913	0.412052	newly	303.	632.866	{96., 1229.}	139.87	0.391417	Reff	2.5648	2.72876	{2.42166, 2.96012}	9.99996	ar	90302.	91719.9	{88414., 93958.}	3.09033
4	R0	{2.5856, 9.28508}	{2.14818, 7.11771}	{{1.37974, 3.3318}, {2.87797, 10.9639}}	{21.5724, 31.8668}	inf	8.	15.0167	{1., 31.}	138.542	0.511652	newly	20.	32.8833	{1., 67.}	127.583	0.595455	Reff	2.58508	2.26267	{1.50257, 2.9355}	18.0401	ar	90312.	69318.4	{6., 93485.}	24.5643
5	R0	{2.4406, 12.1341}	{1.23052, 2.34549}	{{1.19264, 2.01465}, {1.31023, 3.04996}}	{49.5813, 80.6703}	inf	184.	239.333	{0., 580.}	112.319	0.969769	newly	170.	940.278	{0., 2776.}	505.	1.15835	Reff	2.43279	1.22746	{1.15895, 1.34862}	49.5452	ar	88597.	27466.2	{69., 44436.}	68.9988
6	R0	{2.72155, 15.4094}	{2.25768, 11.455}	{{1.88486, 8.39294}, {2.80057, 16.2348}}	{18.8793, 28.4127}	inf	411.	1678.9	{244., 4073.}	324.859	0.533702	newly	302.	1432.88	{186., 3093.}	388.017	0.578036	Reff	2.7028	2.27638	{1.78787, 2.79923}	17.6132	ar	91682.	84933.9	{75786., 93390.}	8.06483
7	R0	{2.63781, 14.3935}	{2.57863, 17.6823}	{{2.28144, 14.5901}, {2.79269, 19.898}}	{8.21202, 24.8331}	inf	448.	429.91	{72., 1048.}	65.1386	0.351066	newly	307.	236.791	{51., 569.}	57.7374	0.374426	Reff	2.61787	2.571	{2.23743, 2.78387}	8.1783	ar	91192.	89891.6	{85834., 92614.}	2.93604
8	R0	{2.82391, 16.104}	{2.37552, 12.1735}	{{1.93419, 8.56054}, {2.76203, 15.3124}}	{16.275, 25.1158}	inf	560.	1083.67	{123., 2247.}	136.682	0.495335	newly	385.	927.688	{102., 2352.}	181.039	0.528349	Reff	2.79954	2.38872	{1.90131, 2.74281}	14.9537	ar	92664.	86089.8	{77893., 92172.}	7.20473
9	R0	{2.80645, 14.6457}	{2.32823, 13.4761}	{{1.68297, 7.5059}, {2.86501, 18.4275}}	{18.1977, 24.9643}	inf	226.	183.067	{32., 404.}	56.1947	0.36579	newly	148.	139.817	{28., 319.}	61.6104	0.346666	Reff	2.79573	2.39999	{1.86431, 2.87173}	15.6789	ar	92567.	85790.8	{75831., 93361.}	7.69603
10	R0	{1.86213, 5.4271}	{2.43244, 7.76368}	{{1.98552, 5.56151}, {2.9194, 10.1535}}	{32.4432, 47.8707}	inf	32.	79.4063	{10., 161.}	178.223	0.466196	newly	74.	145.031	{18., 263.}	132.39	0.450682	Reff	1.8607	2.47383	{2.02782, 2.91155}	33.9458	ar	75362.	84815.6	{79410., 93441.}	19.8283
11	R0	{2.27349, 14.6338}	{1.30094, 3.11158}	{{1.30094, 3.11158}, {1.30094, 3.11158}}	{42.7778, 78.737}	inf	1278.	1390.82	{119., 2953.}	64.218	0.352402	newly	843.	3588.47	{326., 7065.}	343.026	0.625259	Reff	2.22036	1.20035	{1.06993, 1.29352}	45.9391	ar	85805.	42208.9	{41355., 43623.}	50.8083
12	R0	{2.77261, 14.1894}	{2.25981, 15.9815}	{{2.01902, 13.0262}, {2.52006, 19.5955}}	{18.4951, 17.8624}	inf	206.	144.593	{16., 343.}	60.3011	0.463136	newly	178.	84.9492	{10., 215.}	63.4927	0.582119	Reff	2.76385	2.25646	{2.02245, 2.63945}	18.358	ar	92508.	84993.6	{80490., 90854.}	8.12298
13	R0	{2.65402, 19.3935}	{2.06028, 11.654}	{{1.87927, 9.84456}, {2.40085, 15.146}}	{22.3713, 39.9077}	inf	1354.	2872.32	{585., 6038.}	138.454	0.402268	newly	658.	2219.9	{487., 4460.}	253.066	0.521321	Reff	2.59651	1.9549	{1.72861, 2.31318}	24.7105	ar	91184.	80577.2	{75688., 88094.}	11.6323
14	R0	{1.75297, 4.45354}	{1.07082, 1.86744}	{{1.06836, 1.81005}, {1.06836, 2.14605}}	{38.9141, 58.0685}	inf	18.	49.8182	{14., 83.}	191.919	0.430697	newly	29.	236.091	{79., 364.}	723.511	0.836434	Reff	1.75187	1.06513	{1.05822, 1.06724}	39.2003	ar	71263.	10944.6	{427., 15911.}	84.6419
15	R0	{1.99928, 7.1501}	{2.10804, 9.1277}	{{1.49267, 4.47101}, {2.86193, 14.6465}}	{22.3604, 51.0119}	inf	97.	117.492	{14., 281.}	89.0617	0.474317	newly	168.	159.661	{11., 436.}	82.5868	0.561209	Reff	1.99548	2.2079	{1.49847, 2.95271}	22.4215	ar	79602.	78950.9	{59057., 93909.}	15.1532
16	R0	{2.67398, 10.3134}	{2.14926, 13.8695}	{{1.71608, 9.06807}, {2.76849, 18.7697}}	{20.8657, 41.6465}	inf	44.	53.9298	{4., 138.}	91.866	0.50181	newly	54.	35.5965	{3., 77.}	65.0747	0.620837	Reff	2.67219	2.26291	{1.79637, 2.788}	17.006	ar	91289.	81171.5	{71703., 92629.}	11.5557
17	R0	{2.44634, 16.3226}	{2.59268, 16.777}	{{2.33995, 14.464}, {2.64334, 17.2462}}	{7.33568, 6.29588}	inf	1288.	1206.8	{92., 2876.}	70.2126	0.464753	newly	777.	688.677	{58., 1638.}	67.0132	0.469594	Reff	2.39443	2.55371	{2.45195, 2.63937}	7.4522	ar	88710.	90466.1	{89160., 91155.}	2.3221
18	R0	{2.02168, 10.1676}	{2.60072, 17.475}	{{2.11132, 12.644}, {2.86095, 19.9929}}	{29.2969, 71.9198}	inf	354.	512.174	{79., 1482.}	89.1182	0.3718	newly	351.	296.841	{39., 760.}	63.6979	0.440991	Reff	2.00733	2.59243	{2.27626, 2.84928}	29.6457	ar	80299.	90036.2	{86297., 93077.}	12.4175
19	R0	{2.89969, 15.009}	{2.60515, 16.7391}	{{2.21449, 13.2892}, {2.89334, 18.9813}}	{10.3179, 17.6752}	inf	224.	184.591	{39., 529.}	67.0657	0.398736	newly	179.	110.955	{26., 277.}	63.4586	0.456649	Reff	2.88972	2.59003	{2.0459, 2.88912}	10.5509	ar	93349.	89640.2	{81045., 93204.}	4.01871
20	R0	{2.68933, 7.34928}	{2.11828, 11.1354}	{{1.29927, 4.24222}, {2.78007, 17.7348}}	{23.0074, 66.5747}	inf	3.	8.04688	{0., 20.}	222.396	0.619483	newly	7.	8.89063	{0., 28.}	120.759	0.780586	Reff	2.68909	2.20293	{1.39878, 2.85646}	20.2922	ar	91574.	65247.4	{2., 92579.}	29.2069
21	R0	{2.15352, 9.28074}	{1.90954, 7.22975}	{{1.42949, 4.00571}, {2.71225, 12.7047}}	{24.8446, 41.4272}	inf	131.	181.216	{18., 350.}	92.0072	0.450557	newly	151.	296.471	{26., 732.}	144.488	0.510677	Reff	2.14827	2.07506	{1.48488, 2.70763}	22.6513	ar	83845.	73257.	{54487., 91753.}	19.3949
22	R0	{2.21953, 9.04387}	{1.76303, 5.84544}	{{1.24751, 2.5364}, {2.48617, 10.2848}}	{27.5023, 45.0638}	inf	114.	96.5682	{6., 217.}	70.7137	0.543478	newly	136.	224.795	{9., 629.}	137.283	0.590329	Reff	2.21565	1.87836	{1.3094, 2.52134}	23.882	ar	84856.	66893.2	{42729., 89462.}	24.4203
23	R0	{2.21987, 14.5347}	{2.24293, 14.275}	{{1.77826, 9.06807}, {2.53519, 17.5902}}	{9.96443, 15.9232}	inf	1324.	2441.34	{429., 5927.}	120.519	0.379867	newly	822.	1621.78	{250., 3994.}	130.506	0.369709	Reff	2.16759	2.15828	{1.73275, 2.51609}	9.76893	ar	85067.	84537.5	{72517., 92023.}	4.63719
24	R0	{2.84268, 17.9659}	{2.20225, 16.0241}	{{1.88607, 11.9453}, {2.45321, 19.3027}}	{22.5291, 14.9909}	inf	799.	1089.05	{162., 2255.}	86.4059	0.356358	newly	439.	631.267	{94., 1416.}	92.5361	0.361959	Reff	2.80769	2.15906	{1.82632, 2.42591}	23.1017	ar	92836.	83740.1	{76339., 89095.}	9.79787
25	R0	{2.52706, 10.3679}	{2.05672, 15.2904}	{{1.63746, 10.1332}, {2.42205, 19.5287}}	{18.6122, 51.8698}	inf	97.	91.5273	{16., 212.}	62.0244	0.367188	newly	97.	54.9455	{9., 122.}	64.8735	0.554298	Reff	2.52279	2.12511	{1.81059, 2.41584}	15.7636	ar	89623.	81613.3	{73741., 87988.}	8.93713
26	R0	{2.62835, 19.1088}	{2.06037, 11.1732}	{{1.93816, 10.0503}, {2.68803, 17.202}}	{23.1165, 41.62}	inf	1699.	2232.42	{242., 4547.}	89.1416	0.401871	newly	814.	1888.89	{226., 4047.}	164.863	0.427714	Reff	2.55757	1.98691	{1.73763, 2.66381}	23.9466	ar	90908.	80548.3	{77686., 91840.}	11.8975
27	R0	{2.76831, 19.4155}	{1.61772, 5.93387}	{{1.54465, 5.30296}, {1.95355, 8.88568}}	{41.5627, 69.4375}	inf	2186.	4490.88	{807., 8366.}	134.853	0.369594	newly	1084.	5352.53	{1524., 9727.}	397.292	0.624673	Reff	2.67358	1.48496	{1.09854, 1.85525}	44.458	ar	92175.	67290.5	{60587., 87261.}	26.997
28	R0	{2.96609, 16.8903}	{2.2528, 8.1467}	{{1.61691, 4.41442}, {2.91946, 11.8546}}	{24.1094, 51.767}	inf	591.	1166.72	{218., 2600.}	132.941	0.40011	newly	383.	1660.66	{284., 3894.}	349.337	0.577832	Reff	2.94132	2.2993	{1.8047, 2.82344}	21.8748	ar	94024.	84420.5	{74539., 93452.}	10.2187
29	R0	{2.17046, 14.39}	{1.44487, 5.52388}	{{1.42659, 5.32185}, {1.42659, 5.32185}}	{33.4302, 61.613}	inf	1169.	1486.85	{354., 2833.}	69.2203	0.273574	newly	743.	2311.07	{453., 4355.}	220.418	0.439907	Reff	2.12193	1.38276	{1.27283, 1.65751}	34.8347	ar	84001.	55338.7	{52858., 69053.}	34.1214
30	R0	{2.88772, 12.4647}	{1.79668, 4.05467}	{{1.31246, 2.23442}, {2.45236, 6.2492}}	{37.8624, 67.4708}	inf	43.	120.632	{0., 272.}	228.845	0.818823	newly	59.	474.93	{0., 1246.}	741.897	1.09473	Reff	2.88584	1.90356	{1.31236, 2.48191}	34.0381	ar	93273.	61437.3	{6., 88648.}	34.1318
31	R0	{2.46831, 8.14308}	{2.0357, 10.7496}	{{1.17434, 5.03398}, {2.7943, 18.2127}}	{23.3881, 56.2052}	inf	13.	3.3	{0., 9.}	83.3846	1.14187	newly	16.	6.85	{0., 13.}	100.938	1.39246	Reff	2.46792	2.03559	{1.1743, 2.7941}	23.3848	ar	88762.	40929.2	{1., 92228.}	55.5025
32	R0	{1.93236, 7.38987}	{2.5714, 9.17686}	{{1.98388, 6.25659}, {2.98791, 11.317}}	{34.0378, 29.8085}	inf	96.	191.098	{40., 472.}	129.184	0.3525	newly	158.	297.213	{53., 638.}	125.731	0.349659	Reff	1.92828	2.59176	{2.20304, 2.93462}	35.0602	ar	77654.	89425.8	{84479., 93649.}	15.836
33	R0	{2.82251, 17.6954}	{1.44268, 3.81947}	{{1.36035, 3.22945}, {1.50263, 4.22172}}	{48.8866, 78.4154}	inf	586.	881.154	{31., 2362.}	107.942	0.53117	newly	325.	2216.69	{104., 5780.}	602.02	0.81006	Reff	2.7977	1.43291	{1.31033, 1.63518}	48.7826	ar	92745.	54047.4	{47612., 66693.}	41.7248
34	R0	{1.87083, 12.3994}	{1.92048, 10.248}	{{1.87927, 9.84456}, {1.88096, 9.84456}}	{2.65386, 20.2244}	inf	1336.	2960.25	{274., 6019.}	154.62	0.453821	newly	975.	2578.2	{268., 5269.}	191.251	0.482406	Reff	1.81905	1.79894	{1.5924, 1.90206}	6.80526	ar	76282.	77203.7	{75650., 78885.}	1.81124
35	R0	{2.95779, 16.3003}	{2.44861, 12.6485}	{{1.93419, 8.56054}, {2.86118, 15.5722}}	{17.2149, 22.697}	inf	233.	489.567	{83., 1016.}	142.818	0.424118	newly	159.	403.533	{60., 782.}	177.358	0.438981	Reff	2.94682	2.43757	{1.94917, 2.82556}	17.2813	ar	93873.	86197.2	{77811., 93063.}	8.17683
36	R0	{2.18718, 14.261}	{1.31023, 3.04996}	{{1.31023, 3.04996}, {1.31023, 3.04996}}	{40.095, 78.6133}	inf	1258.	2040.24	{784., 3084.}	85.6575	0.287123	newly	857.	5097.81	{2485., 7104.}	495.899	0.725998	Reff	2.13862	1.14028	{0.988595, 1.26592}	46.6816	ar	84379.	43460.7	{42298., 44573.}	48.4935
37	R0	{2.44912, 9.31118}	{2.25474, 7.73681}	{{1.47608, 3.72349}, {2.91001, 10.9639}}	{17.8497, 26.9096}	inf	41.	50.	{5., 103.}	83.9519	0.438682	newly	54.	79.0145	{7., 193.}	103.328	0.464239	Reff	2.44728	2.36197	{1.61296, 2.92325}	15.5146	ar	88442.	80653.7	{53479., 93574.}	12.52
38	R0	{2.05949, 5.72514}	{1.14642, 1.88465}	{{1.12171, 1.78492}, {1.19264, 2.01465}}	{44.3347, 67.0811}	inf	11.	37.8636	{0., 60.}	307.025	0.888459	newly	26.	189.773	{0., 330.}	681.643	1.14511	Reff	2.05842	1.15227	{1.10433, 1.19214}	44.0218	ar	81587.	15139.1	{42., 31405.}	81.4442
39	R0	{1.75505, 8.52404}	{2.49279, 14.9274}	{{2.02235, 10.6804}, {2.93488, 18.9272}}	{42.2201, 75.7014}	inf	489.	970.154	{190., 2386.}	128.718	0.343876	newly	574.	667.092	{126., 1491.}	77.5315	0.348029	Reff	1.73539	2.50582	{2.15498, 2.90571}	44.8866	ar	70925.	88756.6	{83936., 93622.}	25.3674
40	R0	{2.24724, 13.7861}	{1.31023, 3.04996}	{{1.31023, 3.04996}, {1.31023, 3.04996}}	{41.696, 77.8766}	inf	1281.	1000.33	{199., 2050.}	58.3324	0.406834	newly	906.	2889.43	{666., 5646.}	238.095	0.475164	Reff	2.19627	1.24612	{1.16751, 1.29967}	43.2622	ar	85257.	43460.7	{42298., 44573.}	49.0239
41	R0	{1.77635, 8.62406}	{1.36264, 3.24619}	{{1.36035, 3.22945}, {1.36035, 3.22945}}	{23.2899, 62.3589}	inf	634.	955.4	{131., 1876.}	96.4353	0.35472	newly	682.	2773.6	{445., 5456.}	317.185	0.529973	Reff	1.75212	1.31378	{1.22672, 1.3528}	25.0178	ar	72941.	48302.1	{46157., 49009.}	33.7792
42	R0	{1.80397, 5.4932}	{1.84105, 4.89239}	{{1.31246, 2.23442}, {2.68628, 8.57964}}	{24.7523, 42.4507}	inf	36.	230.184	{17., 485.}	559.807	0.767196	newly	83.	650.898	{51., 1452.}	702.557	0.851186	Reff	1.80242	1.9542	{1.31221, 2.83755}	27.2931	ar	73232.	69322.5	{42123., 93114.}	25.2896
43	R0	{2.83096, 16.7077}	{2.44704, 18.547}	{{2.22867, 15.6411}, {2.52006, 19.5955}}	{13.5615, 13.6686}	inf	491.	347.279	{56., 760.}	56.8138	0.402598	newly	311.	177.191	{30., 386.}	58.5918	0.458957	Reff	2.80905	2.42442	{2.20852, 2.50559}	13.6926	ar	92740.	88240.7	{84887., 89516.}	4.85148
44	R0	{2.73678, 11.6021}	{2.50247, 9.90234}	{{2.19024, 8.12562}, {2.96796, 12.6436}}	{11.7411, 17.967}	inf	128.	338.91	{39., 858.}	195.487	0.449598	newly	125.	435.791	{45., 1026.}	273.11	0.505286	Reff	2.73147	2.50307	{2.13818, 2.93332}	11.1903	ar	92003.	88519.3	{84072., 93679.}	4.5159
45	R0	{2.14235, 6.98601}	{2.32023, 15.2612}	{{1.88056, 11.3627}, {2.78218, 19.8588}}	{15.3914, 120.376}	inf	49.	26.6923	{4., 59.}	59.5918	0.519074	newly	74.	17.4769	{2., 33.}	78.6279	0.90895	Reff	2.14079	2.37267	{1.99814, 2.81246}	15.4338	ar	82974.	84531.8	{78469., 92467.}	9.18391
46	R0	{2.23409, 9.21289}	{2.5412, 14.7643}	{{1.95791, 9.82151}, {2.88523, 18.2127}}	{18.8498, 62.5816}	inf	58.	59.4769	{9., 124.}	70.2918	0.360465	newly	78.	41.5077	{4., 99.}	64.2604	0.535305	Reff	2.23183	2.57749	{1.96532, 2.90029}	20.0346	ar	85163.	88850.2	{78666., 93232.}	8.10953
47	R0	{2.12258, 8.15265}	{2.4053, 7.07831}	{{1.88643, 4.56746}, {2.87797, 9.24469}}	{19.0875, 20.362}	inf	73.	110.068	{14., 229.}	95.2171	0.430348	newly	100.	252.068	{29., 556.}	180.847	0.50192	Reff	2.11978	2.37612	{1.82174, 2.82727}	17.7518	ar	82690.	82839.4	{73869., 92846.}	11.0706
48	R0	{2.71771, 10.4636}	{2.3172, 8.89031}	{{1.5451, 4.79544}, {2.87181, 12.4643}}	{17.3519, 23.3675}	inf	20.	37.1719	{4., 94.}	131.328	0.426385	newly	23.	56.1094	{4., 128.}	184.307	0.502493	Reff	2.71654	2.43757	{1.75152, 2.93484}	13.4833	ar	91716.	83386.1	{70897., 93620.}	9.88464
49	R0	{2.44369, 6.66477}	{2.15114, 5.08361}	{{1.27121, 2.14605}, {2.85474, 9.2843}}	{20.717, 44.9863}	inf	7.	6.67	{0., 21.}	108.429	0.995938	newly	14.	42.04	{0., 140.}	308.286	1.25702	Reff	2.44335	2.15088	{1.27117, 2.8524}	20.7154	ar	88827.	43842.5	{1., 92520.}	52.179
50	R0	{2.77383, 17.9775}	{2.47241, 15.701}	{{2.29055, 13.7413}, {2.64334, 17.2462}}	{10.9293, 12.887}	inf	1401.	1236.91	{255., 2817.}	61.9749	0.353469	newly	799.	754.03	{150., 1557.}	63.4394	0.345176	Reff	2.71353	2.44099	{2.25014, 2.60911}	10.1803	ar	92334.	88980.4	{86034., 90981.}	3.64953
